# Structure-Function Analysis of the C-clamp of TCF/Pangolin in Wnt/ß-catenin Signaling

**DOI:** 10.1371/journal.pone.0086180

**Published:** 2014-01-20

**Authors:** Aditi Ravindranath, Ken M. Cadigan

**Affiliations:** Department of Molecular, Cellular and Developmental Biology, University of Michigan, Ann Arbor, Michigan, United States of America; Simon Fraser University, Canada

## Abstract

The evolutionarily conserved Wnt/ß-catenin (Wnt/ß-cat) pathway plays an important role in animal development in metazoans. Many Wnt targets are regulated by members of the TCF/LEF1 (TCF) family of transcription factors. All TCFs contain a High Mobility Group (HMG) domain that bind specific DNA sequences. Invertebrate TCFs and some vertebrate TCF isoforms also contain another domain, called the C-clamp, which allows TCFs to recognize an additional DNA motif known as the Helper site. While the C-clamp has been shown to be important for regulating several Wnt reporter genes in cell culture, its physiological role in regulating Wnt targets is less clear. In addition, little is known about this domain, except that two of the four conserved cysteines are functionally important. Here, we carried out a systematic mutagenesis and functional analysis of the C-clamp from the *Drosophila* TCF/Pangolin (TCF/Pan) protein. We found that the C-clamp is a zinc-binding domain that is sufficient for binding to the Helper site. In addition to this DNA-binding activity, the C-clamp also inhibits the HMG domain from binding its cognate DNA site. Point mutations were identified that specifically affected DNA-binding or reduced the inhibitory effect. These mutants were characterized in TCF/Pan rescue assays. The specific DNA-binding activity of the C-clamp was essential for TCF/Pan function in cell culture and in patterning the embryonic epidermis of *Drosophila*, demonstrating the importance of this C-clamp activity in regulating Wnt target gene expression. In contrast, the inhibitory mutation had a subtle effect in cell culture and no effect on *TCF/Pan* activity in embryos. These results provide important information about the functional domains of the C-clamp, and highlight its importance for Wnt/ß-cat signaling in *Drosophila*.

## Introduction

The Wnt/ß-catenin (Wnt/ß-cat) pathway is a major cell-cell signaling pathway found throughout metazoans [1]. This signaling cascade plays many important roles in animal development [1], [2], [3], [4]. For example, in *Drosophila*, the pathway is important for many cell specification events, including patterning of the embryonic epidermis [5], [6]. Wnt/ß-cat signaling is also critical for adult tissue homeostasis, where it often functions as a stem cell niche signal [7], [8]. Misregulation of the pathway is implicated in many diseases in humans, including many cancers [7], [9], bone disorders [10] and type II diabetes [11].

A key intracellular messenger in Wnt/ß-cat signaling is ß-catenin, whose degradation is inhibited by Wnt stimulation [12], [13]. Stabilized ß-catenin translocates from the cytoplasm to the nucleus, where it is recruited to Wnt target gene chromatin by binding to transcription factors [1], [13]. Members of the TCF/LEF1 (TCF) family are the best-characterized nuclear mediators of Wnt/ß-cat signaling [14], [15]. Vertebrates contain several *TCF* genes, while *Drosophila* has only one, *TCF/Pangolin* (*TCF/Pan*) [16], [17].

All TCFs contain High Mobility Group (HMG) domains, which can bind DNA with sequence specificity [14]. Consensus HMG binding sites are 9–11 bp in length, and share the sequence SCTTTGWWS [17], [18], [19]. Synthetic reporters comprised of multimerized consensus HMG sites upstream of a basal promoter can be activated by Wnt/ß-cat signaling [20] and functional high affinity HMG sites are found in many endogenous Wnt/ß-cat regulated cis-regulatory modules (W-CRMs) [1]. In addition to these high affinity HMG sites, the HMG domains of TCFs can bind numerous lower-affinity secondary sites [21], some of which have been shown to be functional [22], [23]. Because of the degeneracy of HMG-DNA recognition, it seems unlikely that these interactions are sufficient for TCFs to locate their nuclear targets [1].

Several TCFs increase their DNA binding specificity through a second DNA binding domain known as the C-clamp. This domain, located just C-terminal to the HMG domain, was originally discovered in “E-tail isoforms” of vertebrate TCF1 and TCF4 [24]. C-clamps are also found in nearly all invertebrate TCFs, including TCF/Pan [1], [14]. The C-clamp is required for TCFs to bind to a second DNA motif known as the Helper site, which is critical for Wnt activation of several fly and human W-CRMs [25], [26]. The working model is that C-clamp containing TCFs recognize DNA through a combination of HMG domain-HMG site and C-clamp-Helper site interactions [14]. The C-clamp containing TCF1E and TCF4E isoforms have been implicated in promoting colorectal cancer [24], [26] and regulating Wnt targets in embryonic stem cells [27].

In addition to the TCF1E and TCF4E isoforms, there are other C-clamp containing proteins in humans. The best characterized is known as SLC2A4 regulator (SLC2A4RG), GLUT4 enhancer factor (GLUT4EF) or Huntington’s disease binding protein 1 (HDBP1), which can bind to the promoters of Huntington’s disease gene [28] and *GLUT4* [29] and is a candidate locus for increased risk to Crohn’s disease and ulcerative colitis [30], [31]. The related protein HDBP2, also known as papilloma virus binding factor (PBF) or zinc finger 395 (ZNF395), represses human papillomavirus virus expression [32], [33], [34] and promotes adipogenesis [34]. The third C-clamp protein, ZNF704 or glucocorticoid induced gene 1 (GIG1) can bind to a *myoD* enhancer [35]. There is one homolog of these genes in *Drosophila*, known as fly *Glut4EF*, which is required for proper wing position in adults [36].

In contrast to the HMG domain, where a structure of the domain bound to a high affinity site has been determined [37], little is known about the structure of the recently discovered C-clamp. Based on sequence alignments, the C-clamp consists of 30 residues [1], [14], [24]. C-clamps contain four conserved cysteines, and limited mutagenesis studies indicate that some are required for function [24]. The C-clamp domains of HDBP1 and HDBP2 are sufficient for specific binding to Helper site-like sequences [28], but for TCFs, specific binding has only been observed in conjunction with the adjacent HMG domain [24], [25]. Further investigation of how these C-clamps recognize DNA is needed to better understand their role in Wnt/ß-cat signaling and other processes.

In this report, we explore the physical properties and functional relevance of the C-clamp through a combination of biochemical and genetic assays. We find that the C-clamp of TCF/Pan is sufficient for binding to DNA containing a Helper site and that the C-clamp contains a zinc ion that is essential for this DNA-binding activity. Site-directed mutagenesis demonstrated that all four cysteines and a stretch of basic residues are essential for specific DNA binding, and the ability to activate W-CRMs reporters in fly cell culture. We also found that the C-clamp can bind to the HMG domain and inhibits its ability to bind to HMG site DNA. A *TCF/Pan* gene containing a point mutation that specifically inhibited this inhibitory activity was compromised for activity in fly cell culture, but was able to rescue the embryonic patterning defect of *TCF/Pan* mutants. However, a DNA-binding mutant had no rescue activity in fly embryos. These data provide important biochemical information about the C-clamp of TCFs, and provide the first direct evidence for the importance of this domain in mediating Wnt/ß-cat target gene regulation in *Drosophila* embryos.

## Results

### The C-clamp is Zinc Ion-Dependent DNA Binding Domain

**Figure 1:**
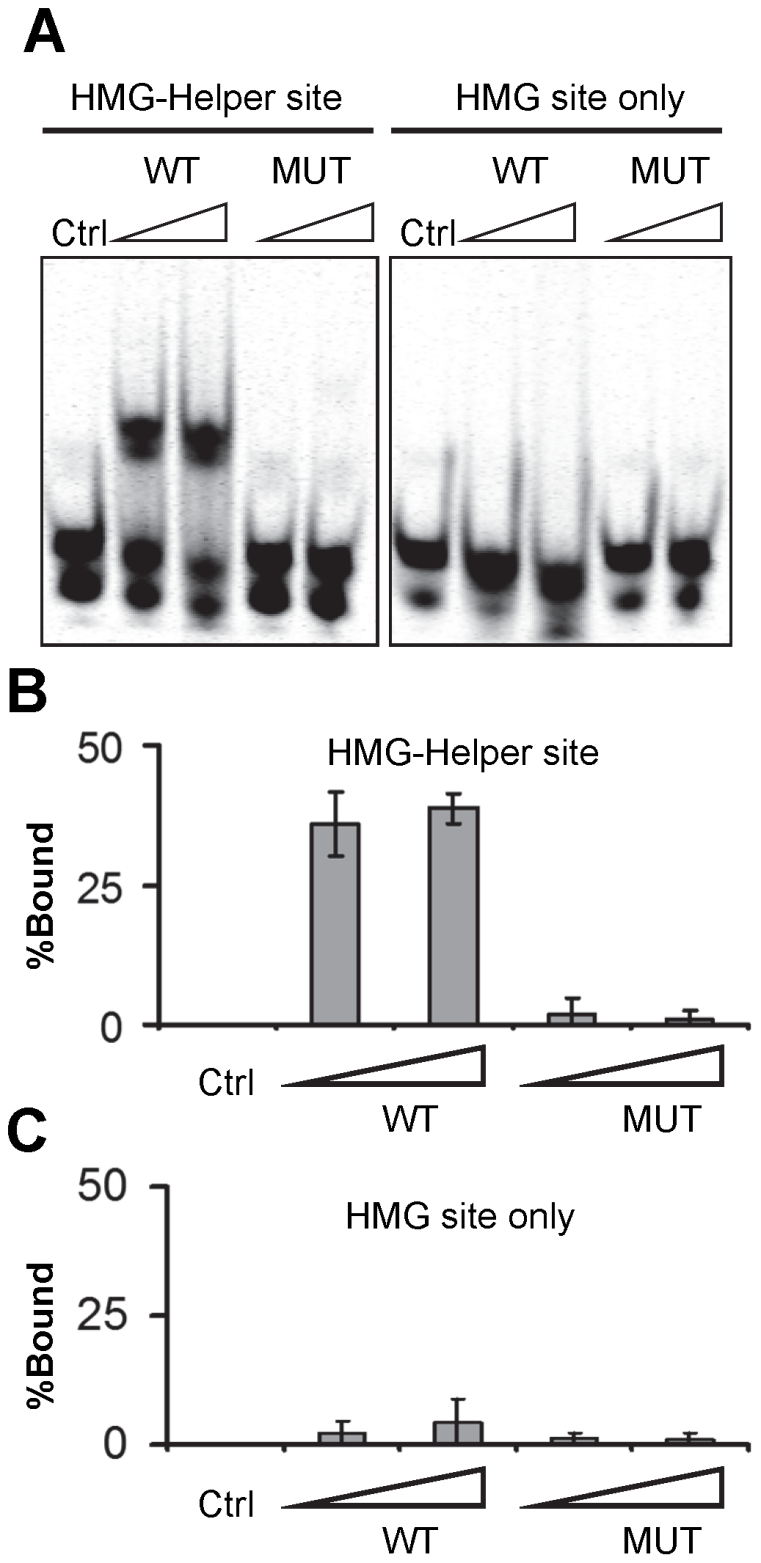
The C-clamp domain is sufficient for binding to the Helper site. (A) EMSA showing that a His-tagged C-clamp domain can bind to a DNA probe containing a Helper site, while a C-clamp protein containing two mutations in the domain (K2A, R4E; same as mutant 5 in Figure 3A) has greatly reduced binding. Neither protein bound a probe lacking a Helper site. For each binding reaction 50 and 100 pmoles of protein and 20 fmoles of oligonucleotide were used. (B,C) Quantification of the EMSA data using the Licor system. The bar graph results are the means of at least three separate binding reactions±SD. See Materials and Methods for details.

Previous studies have shown that the presence of the C-clamp allows the HMG domain of TCF proteins to bind to a bipartite site containing HMG and Helper sites [24], [25], [26]. However, the ability of the C-clamp to bind to the Helper site independently of the HMG domain has not been tested for TCF/Pan [25], while in a human TCF1E isoform, a protein fragment downstream of the HMG domain (containing the C-clamp and additional sequences) only displayed non-specific DNA binding activity [24]. To explore whether the C-clamp of TCF/Pan has an intrinsic ability to bind to the Helper site, *E. coli* was used to purify a His-tagged 45 amino acid fragment of TCF/Pan, containing the C-clamp. This protein can bind to a DNA probe containing a Helper site (Figure 1A, 1B). Mutation of two residues at the N-terminus of the C-clamp (K371A and R373E in the full length TCF/Pan) abolished binding (Figure 1A, 1B) and neither wild-type or mutant protein bound DNA lacking the Helper site. These results demonstrate that the C-clamp of TCF/Pan is sufficient for binding to the Helper site.

**Figure 2:**
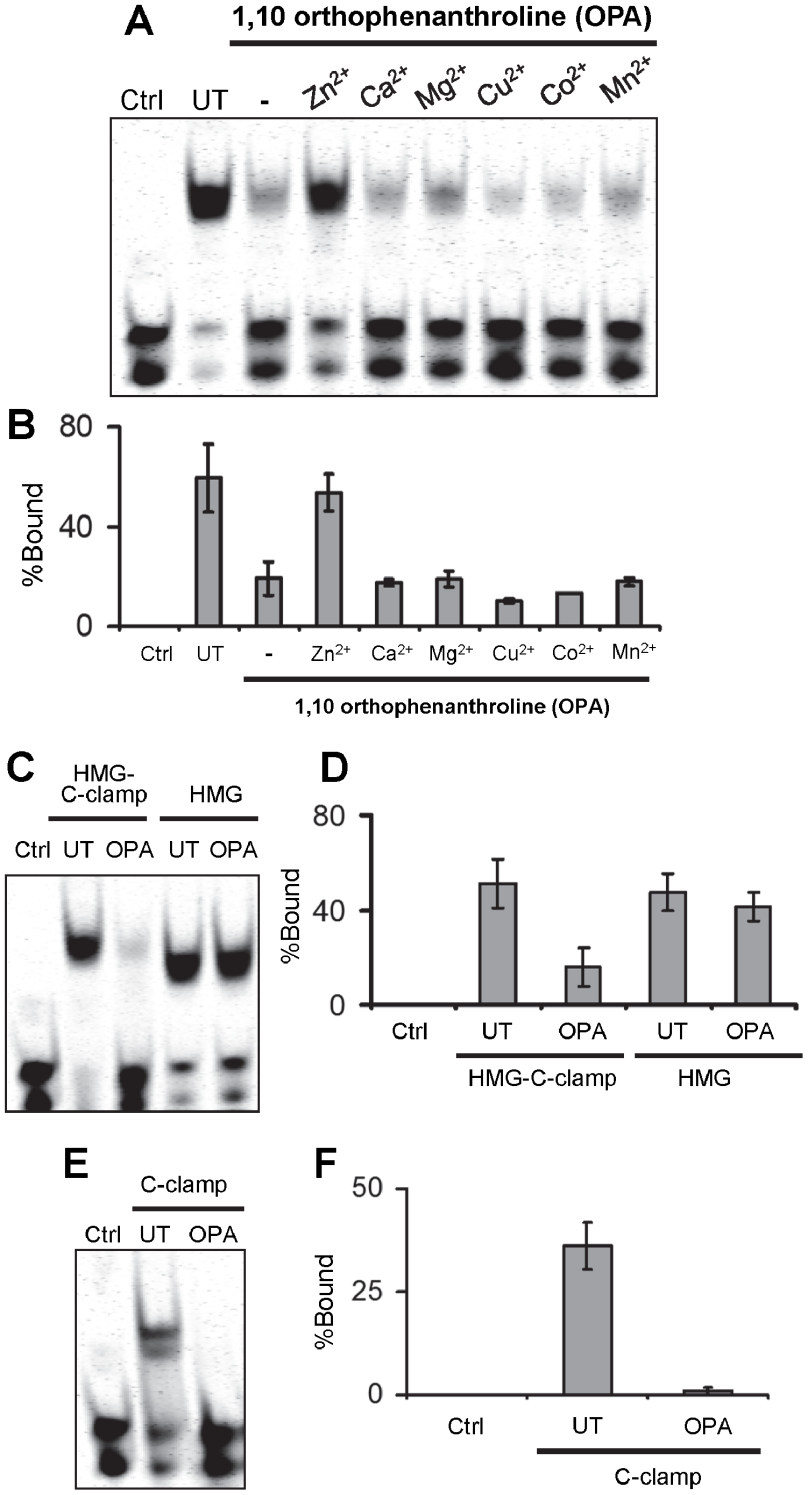
The C-clamp domain requires zinc for binding to the Helper site. (A) Pretreatment of a recombinant fragment of TCF/Pan containing the HMG and C-clamp domains with the metal chelator 1,10-orthophenanthroline (OPA) inhibits its ability to bind to an oligonucleotide containing a HMG and Helper site. Binding was restored by incubation of the OPA-treated protein with zinc but not other divalent metals. Ctrl indicates a probe only lane and UT refers to protein that was untreated by OPA. For each binding reaction 9 pmoles of protein and 20 fmoles of oligonucleotide were used. (B) Licor quantification of the EMSA data. (C, D) EMSA gel and Licor quantification demonstrating that a protein fragment containing only the HMG domain was insensitive to OPA treatment. 9 (HMG-Cclamp) and 12 (HMG only) pmoles of protein and 20 fmoles of oligonucleotide were used. (E, F) EMSA and Licor quantification demonstrating that recombinant C-clamp protein is sensitive to OPA treatment. 50 pmoles of C-clamp protein and 20 fmoles of oligonucleotide were used. All experiments were carried out at least three times and the bar graph results are the means of at least triplicates±SD.

One hallmark of the C-clamp is the presence of four conserved cysteines [14]. This is characteristic of a number of zinc finger domains where four cysteine residues coordinate a zinc ion [38], [39]. To explore the possibility that the C-clamp requires a metal ion, a His-tagged recombinant protein containing the HMG domain and C-clamp from TCF/Pan was treated with metal chelators and subsequently tested for binding to a DNA probe containing a HMG site and a Helper site. Treatment with the metal chelator 1,10-orthophenthroline (OPA) greatly reduced the ability of the HMG-C-clamp protein to bind to this HMG-Helper site probe (Figure 2A–2D). A similar inhibition was observed with EDTA, another metal chelator (data not shown). OPA-treatment also inhibited the ability of recombinant C-clamp protein to bind to the HMG-Helper site probe (Figure 2E, 2F), but had no effect on HMG domain DNA binding (Figure 2C, 2D). These results suggest that the C-clamp contains a metal ion that is critical for its ability to bind the Helper site.

To determine the identity of the specific metal required for C-clamp-dependent DNA binding, OPA treated protein was incubated with several metal ions prior to DNA binding. Of the six divalent metal ions tested (Zn^2+^, Ca^2+^, Mg^2+^, Cu^2+^, Co^2+^ & Mn^2+^), only zinc restored high affinity binding to the HMG-Helper site probe (Figure 2A, 2B). Consistent with these data, inductively coupled plasma mass spectrometry (ICP-MS) detected zinc at near stoichiometric levels in the HMG-C-clamp protein preparation, while only background levels were found in the recombinant HMG domain (Table 1). These results argue strongly that the C-clamp of TCF/Pan contains one molecule of Zn^2+^, whose presence is required for binding to the Helper site.

**Table 1:** Recombinant HMG-C-clamp fragment contains near stoichiometric amounts of Zinc.

Protein	Protein concentration (µM)	Zn^2+^ ion concentration (µM)
HMG-C-clamp	42.1	32.0±1.6
HMG	35.1	<0.4

Recombinant HMG-C-clamp and HMG domain proteins were purified from *E. coli* and subjected to ICP-MS. See Materials and Methods for details of ICP-MS analysis.

### Structure/Function Analysis of the C-clamp in Cultured *Drosophila* Cells

**Figure 3:**
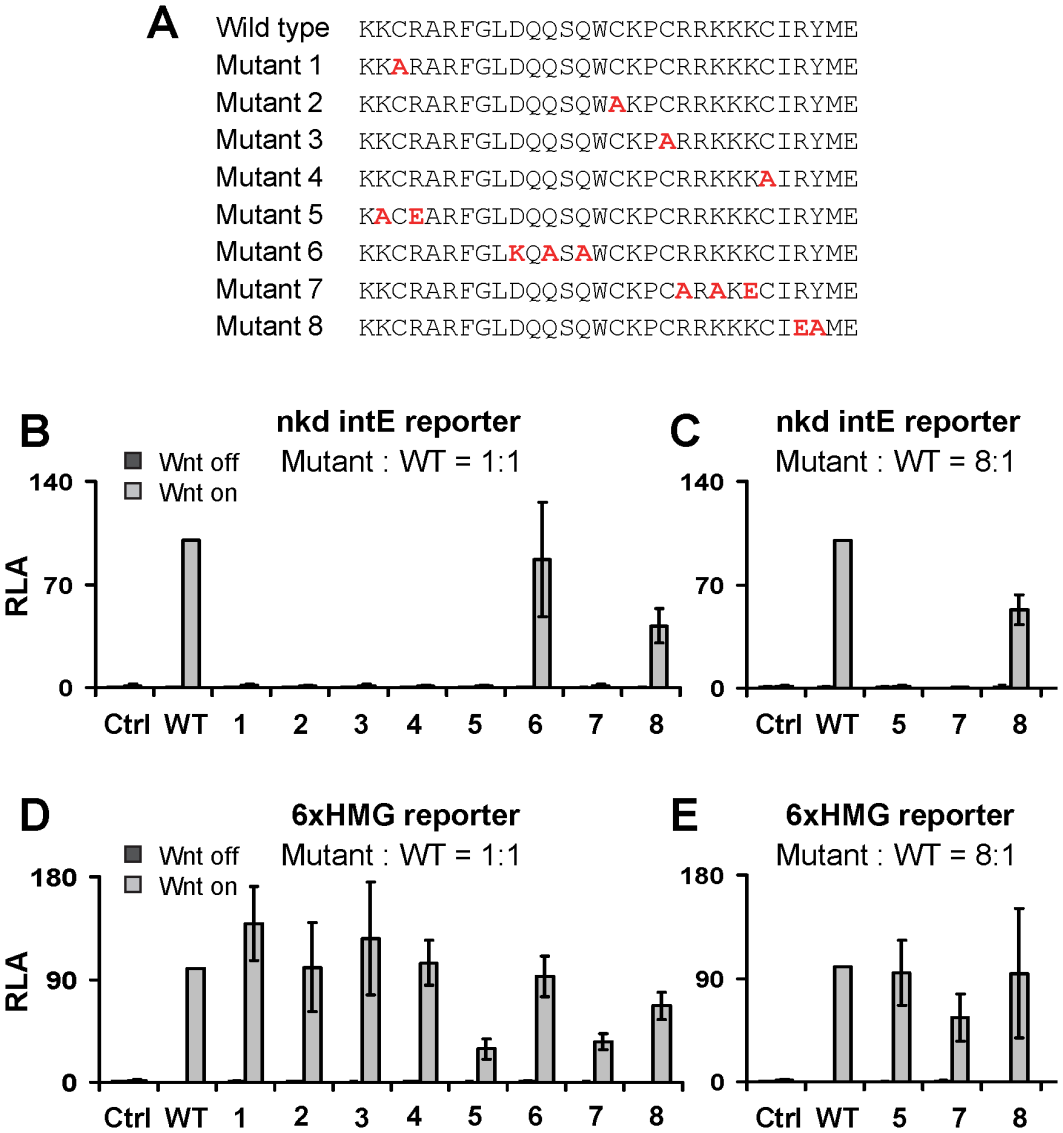
The cysteine and basic residues of the C-clamp are required for activation of a W-CRM reporter in cell culture. (A) Amino acid sequences of the C-clamp in wild-type (WT) TCF/Pan and the eight mutants. The amino acids that have been mutated are in red. (B–E) TCF/Pan RNAi rescue assays carried out in Kc cells. Endogenous TCF/Pan was depleted using dsRNA that targets the TCF 3′ UTR, followed by transient transfection of either WT or C-clamp mutant expression constructs containing a heterologous 3′UTR that cannot be targeted by the dsRNA. The Wnt/ß-cat pathway was induced (Wnt on) using Arm* (see text for further description). Ctrl and “Wnt off” refer to controls where there was transfection of an empty expression vector. The *nkd-IntE* W-CRM reporter was used as an example of a Helper site-dependent W-CRM (B, C), while the synthetic 6xHMG reporter was used as a Helper site-independent Wnt readout (D, E). For some experiments, the mutant TCF/Pan constructs were transfected at eight times the level of wild-type, to ensure that sufficient levels of mutant protein were produced (C, E). Bars are the mean of triplicate transfection SD. Experiments were repeated at least three times with similar results. See Materials and Methods for additional details of the cell culture conditions.

To systematically explore which regions of TCF/Pan’s C-clamp are required for activation of Wnt/ß-cat signaling, eight mutant constructs were generated. These include the four conserved cysteines, which were converted to alanines (Mutants 1–4; Figure 3A). In addition, other highly conserved charged or polar amino acids were converted to alanines or oppositely charged residues (e.g. arginine to glutamic acid) to create TCF/Pan Mutants 5–8 (Figure 3A). These mutants were then tested for their ability to rescue Wnt/ß-cat signaling in *Drosophila* Kc167 (Kc) cells that had been depleted of endogenous TCF/Pan using RNA interference (RNAi) [25].

The readout for Wnt/ß-cat signaling used in the TCF/Pan rescue assays was the *nkd-intE* reporter, which was previously shown to contain functional HMG and Helper sites and to require the C-clamp of TCF/Pan for activation by the pathway in Kc cells [25]. A constitutively active form of Armadillo (the fly ß-cat), referred to as Arm*, was used to activate Wnt/ß-cat signaling in these cells [25], [40], [41], [42]. Almost no detectable activation of the *nkd-intE* reporter was observed in TCF/Pan depleted cells, but expression of wild-type TCF/Pan restored robust activation (Figure 3B). Mutant 6 had similar levels of activity as wild-type TCF/Pan, while Mutant 8 was approximately two-fold less active (Figure 3B). Strikingly, the other six TCF/Pan mutants had no detectable ability to mediate activation of *nkd-intE* by Arm* (Figure 3B).

One caveat with the aforementioned data is that the inactive mutant TCF/Pan proteins may be misfolded or unstable. To address this concern, the wild-type and mutant *TCF/Pan* genes were tagged with the V5 epitope [25], but these proteins could not be detected via Western blot, even when transfected at much higher levels than used in the functional assay and when Kc cells were cultured in the presence of the proteasome inhibitor MG132 (data not shown). Instead, untagged versions of the proteins were tested for their ability to activate a synthetic reporter containing six HMG binding sites upstream of the core promoter/luciferase cassette (6xHMG) [25]. The ability of TCF/Pan to rescue 6xHMG expression served as a proxy to control for expression/activity levels among the various TCF/Pan proteins.

6xHMG was not activated in TCF/Pan depleted Kc cells, but expression of the wild-type protein restored activation (Figure 3D). Mutants 1–4 and 6 showed similar activation as the wild-type (Figure 3D). Mutants 5, 7 and 8 also activated the synthetic reporter, but at lower levels than wild-type (Figure 3D). Increasing amounts of expression construct were transfected and at a ratio of 8∶1, Mutants 5 and 8 could activate 6xHMG at similar levels as wild-type, with Mutant 7 reaching about 60% of the control (Figure 3E). However, even with higher amounts of transfected DNA, Mutants 5 and 7 were unable to activate the *nkd-IntE* reporter, while Mutant 8 topped out at 60% of wild-type (Figure 3C). These data indicate that several residues within the C-clamp are essential for activation of a Helper site-dependent W-CRM in cultured cells but have a minimal effect on the overall stability/folding of TCF/Pan.

### The C-clamp Possesses Two Separate Functions in DNA Recognition

**Figure 4:**
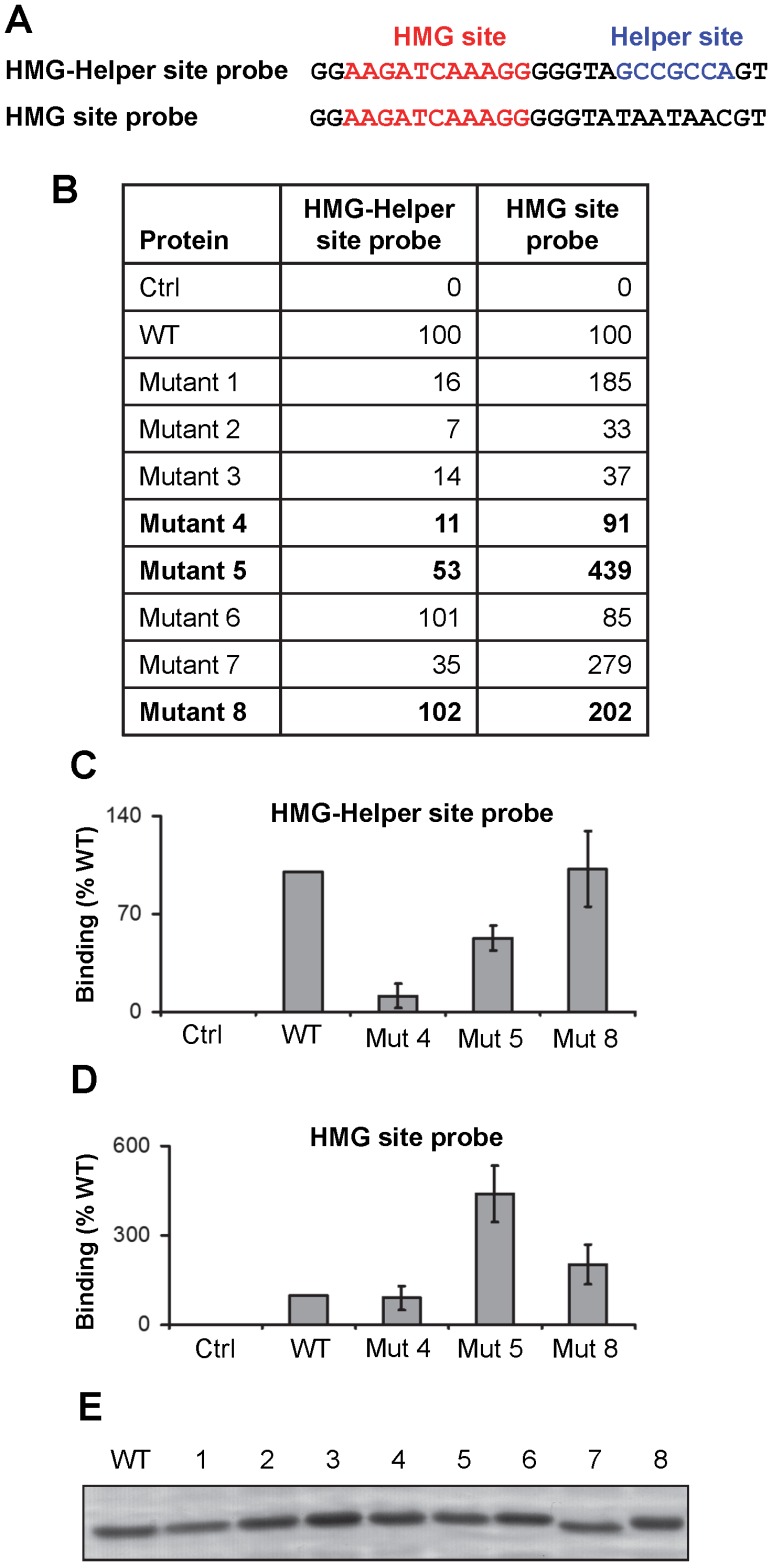
Characterization of the DNA-binding and inhibitory functions of the C-clamp. (A) Sequence of the HMG-Helper site probe and the HMG site oligonucleotide probes used to characterize the ability of C-clamp mutants to bind DNA. (B) Protein fragments containing the HMG domain and wild type (WT) or mutant C-clamps were tested for their ability to bind the HMG-Helper site and the HMG site probes using the Licor EMSA assay described in Figure 1. Ctrl indicates probe only lane. For both probes, WT bound signal was normalized to 100. All mutants were tested at least twice and the averages are reported. Mutants 4, 5 and 8 are in bold, denoting their use in followup experiments. (C, D) EMSA experiments characterizing the defects in Mutants 4, 5 and 8 in binding to the HMG-Helper site (C) and HMG site (D) probes. For each binding reaction, 20 fmol of DNA probe and 9 pmol of protein was used. At these conditions, wild-type protein bound 7–12 times as much HMG-Helper site probe as HMG site probe (data not shown). Data represents means of triplicates±SD. These experiments were repeated three times with similar results. (E) Commassie stained gel of purified WT and C-clamp mutant proteins, demonstrating that each preparation used contained similar amounts of TCF/Pan.

Previous reports and this study have demonstrated that the C-clamp is necessary and sufficient for binding to Helper sites [24], [25] (Figure 1). In addition to this role in DNA binding, there is also published data suggesting that the presence of a functional C-clamp inhibits the ability of the HMG domain to bind to HMG sites [25], [43], [44], [45]. This HMG domain inhibitory function (hereafter referred to as “inhibitory” function) is poorly understood. To explore the DNA-binding and inhibitory functions of the C-clamp in more detail, recombinant HMG-C-clamp fragments of wild-type TCF/Pan and the eight mutants described in Figure 3A were expressed and purified and subjected to quantitative EMSA analysis using probes containing a HMG and Helper site and a probe containing only a HMG site (Figure 4A).

Consistent with our earlier report [25], the recombinant HMG-C-clamp protein bound a HMG-Helper site probe with higher affinity than a HMG site probe (data not shown; see Figure 4 legend). In agreement with previous findings [24], [25], we found that substitutions in any of the four cysteine residues (Mutants 1–4) greatly reduced ability to bind the HMG-Helper site probe (Figure 4B). Mutants 5 and 7 had a moderate reduction in binding the HMG-Helper site probe, while mutants 6 and 8 had comparable binding to wild-type (Figure 4B). When the HMG site probe was tested, several mutants had higher than wild-type binding, indicating a defect in the inhibitory function of the C-clamp, with mutant 5 exhibiting the most dramatic effect (Figure 4B).

Mutants 4, 5 and 8 were selected for further study, since Mutant 4 appeared to have a specific defect in DNA binding activity (i.e., reduced binding to the HMG-Helper site probe; normal binding to the HMG site probe), Mutant 8 appeared to have a specific (though partial) defect in the inhibitory activity (i.e., normal binding to the HMG-Helper site probe; elevated binding to the HMG site probe) and Mutant 5 was defective in both activities. Additional EMSA experiments confirmed these defects (Figure 4C, 4D). Note that Mutant 5 has stronger binding to the HMG-Helper site probe than Mutant 4, presumably due to the conflicting effects of loss of Helper site binding combined with loss of inhibitory activity, which increases binding of the HMG domain to the HMG site in the probe.

**Figure 5:**
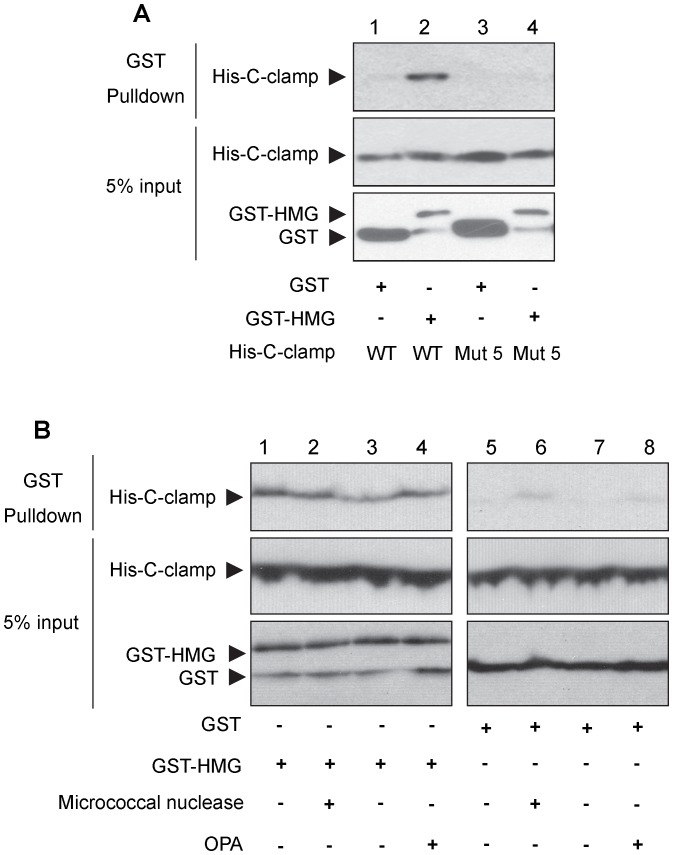
Direct binding of the C-clamp domain to the HMG domain. Western blots using anti-His tag and anti-GST tag antibodies. (A) Pulldown using GST-HMG or the GST control incubated with wild-type (WT) or Mutant 5 His-C-clamp (see Materials and Methods for details of the binding reaction). The upper blot shows an interaction between GST-HMG and the wild-type C-clamp. The middle and lower blots are 5% input of the total reaction mixture. (B) Pulldowns using GST-HMG (lanes 1–4) or GST control (lanes 5–8) and WT His-C-clamp. The proteins were pretreated with micrococcal nuclease (lanes 2 & 6) or OPA (lanes 4 & 8). The negative controls (lanes 1 & 5 for micrococcal nuclease, and lanes 3 & 7 for OPA treatments) were subjected to the same treatment conditions with nuclease free water being used instead of micrococcal nuclease or OPA. The upper blots show the amount of His-C-clamp pulled down and the middle and lower blots are 5% input of the total reaction. All experiments were repeated three times with similar results.

To investigate the mechanism of the C-clamp inhibitory function, the ability of the HMG domain to interact with the C-clamp was tested. GST-tagged HMG domain (GST-HMG) and a His-tagged C-clamp fragment (His-C-clamp) were incubated and analyzed in a GST pulldown assay. GST-HMG binding to His-C-clamp was observed, with minimal binding by the GST negative control (Figure 5A). In contrast, no binding was observed between GST-HMG and the Mutant 5 His-C-clamp (Figure 5A). The HMG-C-clamp interaction was not affected by pre-treatment with micrococcal nuclease, ruling out the possibility that DNA was acting as an adaptor between the two protein domains (Figure 5B). Pretreatment of the C-clamp with the metal chelator OPA also had no effect on binding to the HMG domain, suggesting that coordination of a zinc molecule was not required (Figure 5B). These data suggest that the C-clamp inhibits the ability of the HMG domain to bind its cognate site by direct protein-protein interaction with the HMG domain.

### The C-clamp is Required for Patterning of the Fly Embryonic Epidermis

**Figure 6:**
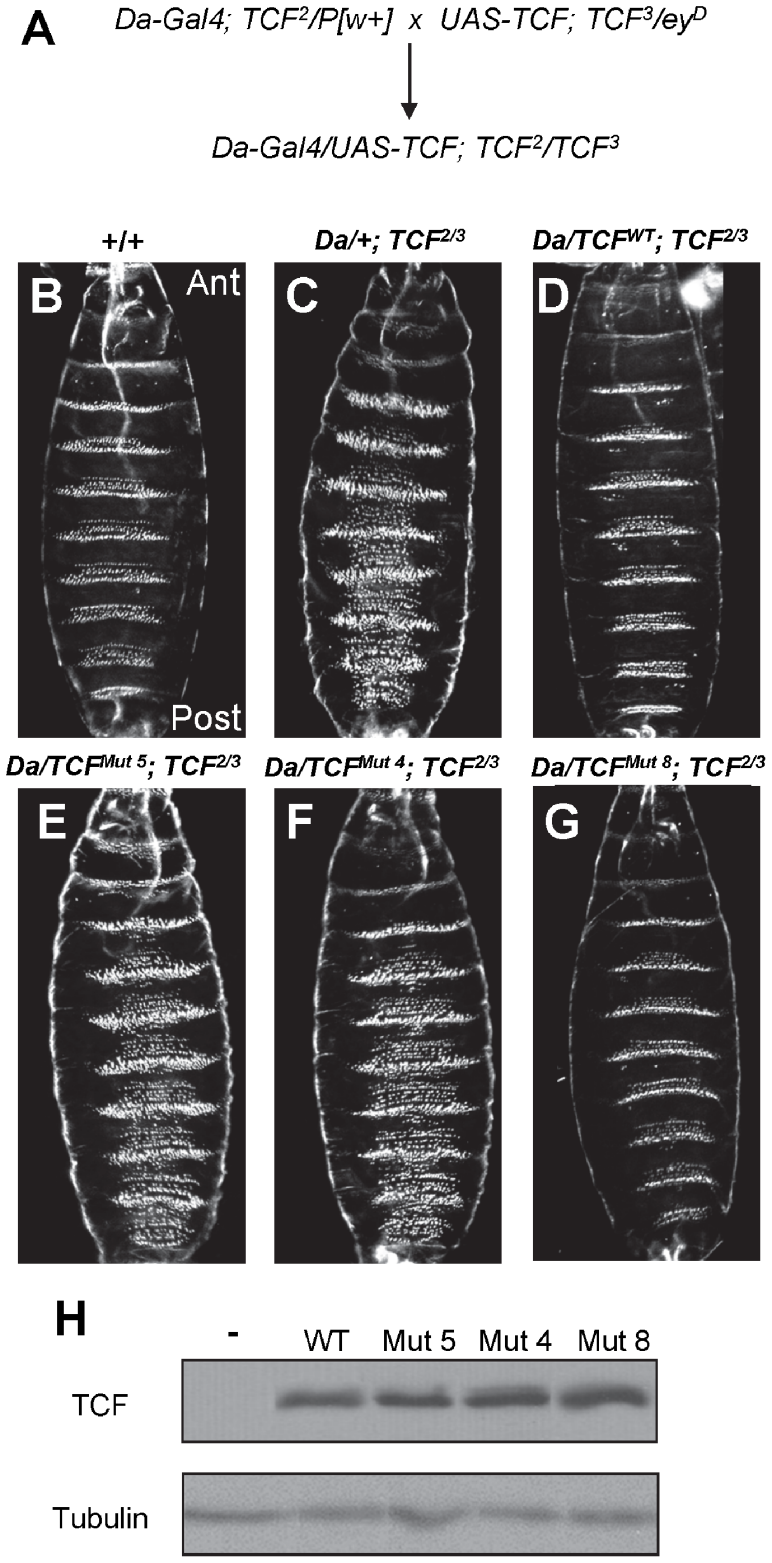
The C-clamp is required for patterning of the *Drosophila* embryonic epidermis. (A) Crossing scheme used to generate embryos containing a P[*Da*-Gal4] driver and P[UAS-*TCF/Pan*] transgene in a *TCF^2^/TCF^3^* transheterozygous mutant background. UAS-*TCF/Pan* (UAS-*TCF*) encodes for either wild type or a mutant *TCF/Pan*. (B–C) Darkfield micrographs of the ventral cuticle control (B) or P[*Da*-Gal4]/+; *TCF^2^/TCF^3^* embryos showing the normal and *TCF/Pan* mutant phenotypes, respectively. (D–G) Cuticles of *TCF^2^/TCF^3^* mutants expressing wild-type (D), mutant 5 (E), mutant 4 (F) or mutant 8 (G) *TCF/Pan* cDNAs. (H) Western blots showing comparable levels of TCF/Pan (upper blot) expression for WT and various C-clamp mutants in P[*Da*-Gal4]; P[UAS-*TCF/Pan*] embryos with Tubulin used as the loading control (lower blot).

To analyze the significance of the DNA binding and inhibitory functions of the C-clamp at the organismal level, transgenic flies with various *TCF/Pan* cDNAs (wild-type and Mutants 4, 5 & 8) under the control of a Gal4-inducible promoter [46] were created using P-element mediated transgenesis [47]. The P[UAS-*TCF/Pan*] transgenes and a *Daughterless*-Gal4 driver (P[*Da*-Gal4]) were crossed into genetic backgrounds containing two independent *TCF/Pan* mutant alleles (*TCF^2^* & *TCF^3^*) believed to be nulls [17]. The *Da*-Gal4 driver is active throughout the embryonic epidermis [48]. *TCF/Pan* transheterozygotes expressing UAS-*TCF/Pan* via the *Da*-Gal4 driver were generated via standard genetic crosses (Figure 6A).

*TCF/Pan* mutants have a strong segment polarity phenotype, which can be visualized with darkfield microscopy of late embryonic cuticles [17], [49]. In control embryos, the anterior portion of each segment contains a trapezoidal array of denticles on the ventral surface, with the posterior portion of the segment displaying naked cuticle lacking denticles (Figure 6B). In *TCF/Pan* mutants, the posterior portion of each segment contains denticles (Figure 6C). Expression of a wild-type *TCF* transgene provides complete or near-complete rescue of this patterning defect with 100% penetrance (Figure 6D; data not shown). Fly embryos expressing either *TCF/Pan* Mutants 4 or 5 had no detectable rescue (Figure 6E, 6F). Surprisingly, Mutant 8 had a level of rescue comparable with that of wild-type *TCF/Pan* (Figure 6G). The P[UAS-*TCF/Pan*] transgenes used in the rescue assay were prescreened for similar levels of expression (Figure 6H).

## Discussion

### The C-clamp is a Zinc-coordinating Motif that is Sufficient to Bind DNA

Previous work demonstrated that the presence of a C-clamp downstream of the HMG domain allowed TCFs to bind to an extended DNA sequence containing a HMG and Helper site [24], [25], [44]. However, a recombinant protein containing the C-clamp had only non-specific DNA binding activity [24], raising some doubt about the nature of the C-clamp-Helper site interaction. Here, we demonstrate that recombinant C-clamp of TCF/Pan specifically binds Helper site DNA (Figure 1). We suspect the difference between our data and Atcha and coworkers is technical. For example, they used a C-terminal fragment of TCF1E containing the C-Clamp plus 95 additional residues (amino acid residues 436 to 561 of human TCF1E). Our positive results are consistent with a report where the C-clamp of GLUT4EF/HDBP1 was sufficient for binding a Helper site-like motif [28].

**Figure 7:**
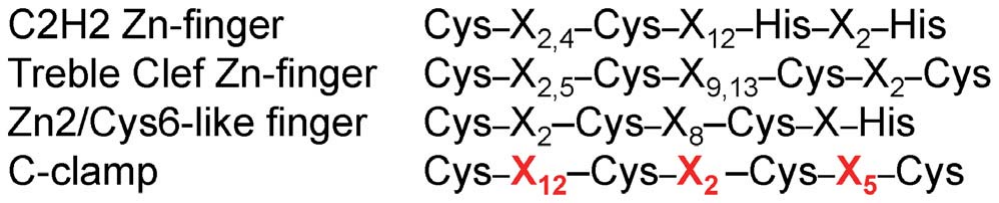
The spacing of cysteine residues in the C-clamp is distinct from other Zn-finger motifs. In a typical C2H2 Zn-finger, the two cysteine and two histidine pairs that coordinate the Zn ion are separated by a stretch of 12 amino acids [38]. In the treble-clef Zn-fingers found in the estrogen and glucocorticoid receptors, two pairs of cysteines are separated by 9 or 13 residues [38], [51]. A similar organization is found in the Zn2/Cys6-like finger of the yeast copper-regulated transcription factor [50]. In contrast, the second and third cysteines of C-clamps are closely paired, with the longest (12 residue) spacing found between the first two cysteines.

Zn-finger motifs contain four residues that coordinate a Zn ion, either all cysteines or a combination of cysteine-histidines [38], [39]. Although C-clamps possess four conserved cysteine residues that are required for DNA binding [24], [25] (Figure 4), it is not readily apparent from the primary sequence that C-clamps are Zn-finger domains. As outlined in Figure 7, C2H2 Zn-fingers and Zn2/Cys6-like fingers (found in many transcription factors) and the Zn tremble clef fingers that form the DNA-binding domain of nuclear receptors have a similar spacing of the coordinating residues [38], [50], [51]. In these DNA-binding Zn domains, a stretch of amino acids (8–13 residues) separates two pairs of closely spaced cysteines or histidines (2–5 residues; Figure 7). In contrast, the conserved spacing of cysteines in C-clamps is distinct (Figure 7). Despite this difference, our results demonstrate that the C-clamp of TCF/Pan requires a Zn ion for its DNA binding activity. Recombinant C-clamp contains near stoichiometric quantities of Zn (Table 1) and metal chelators inhibit Helper site binding (Figure 2). This inhibition was reversed by the addition of Zn^2+^ to the metal-depleted protein (Figure 2A, 2B), providing convincing evidence that the C-clamp is a new class of Zn-finger like domain.

There are two regions between the cysteines of the C-clamp that contain polar or charged amino acids (Figure 3A). Surprisingly, mutation of three charged/polar residues between the first two cysteines (Mutant 6) had no effect on binding to Helper site DNA in vitro (Figure 4B) or the ability of TCF/Pan to activate a Helper site-dependent W-CRM reporter in cultured cells (Figure 3B). However, substitution of three basic residues between the third and fourth cysteines (Mutant 7) greatly reduced Helper site binding in vitro (Figure 4B). *A TCF/Pan* cDNA containing this mutation was incapable of activating the Helper-dependent reporter (Figure 3B, 3C) but was partially able to activate a synthetic HMG site reporter (Figure 3D, 3E). While it is tempting to speculate that these basic residues may make direct contact with the phosphate backbone of the Helper site, structural analysis will be required to more fully understand the nature of the C-clamp-Helper site interaction.

### The DNA Binding Ability of the C-clamp is Functionally Important for Patterning the *Drosophila* Embryo

Helper sites have been shown to be important for activation of several W-CRMs in *Drosophila* tissues [25] and human cell culture [26]. Given the ability of the C-clamp to bind to Helper site DNA [24], [25], [26], [44] (Figure 1), the current model posits that a combination of HMG domain-HMG site and C-clamp-Helper site interactions are required for invertebrate TCFs and mammalian TCF1E and TCF4E isoforms to locate their W-CRM targets [14]. This model is supported by RNAi rescue experiments in fly cell culture demonstrating a requirement for the C-clamp in activating W-CRM reporters [25] (Figure 3B, 3C). In addition, the ability of a dominant negative version of human TCF1E to inhibit growth of a colon cancer cell line requires the C-clamp, suggesting that C-clamp-Helper site interactions are important for Wnt/ß-cat signaling-dependent oncogenesis [24], [26], [44]. Our structure-function analysis of the C-clamp revealed the every C-clamp mutant that had reduced ability to bind to the HMG-Helper site probe (mutants 1–5 & 7; Figure 4B) also had no ability to rescue *nkd IntE* reporter activation in TCF/Pan depleted Kc cells (Figure 3B, 3C). Our results provide strong evidence that the DNA binding activity of the C-clamp is essential for its ability to activate Wnt target genes.

Previously, a *TCF/Pan* allele containing a A374V mutation (the fifth amino acid in the C-clamp) had a weak defect in Wg/Wnt signaling in the *Drosophila* embryo [17]. To more clearly establish the biological relevance of the C-clamp in TCF/Pan function at the organismal level, we established a rescue assay in fly embryos. Null *TCF/Pan* mutants have a strong segment polarity defect [16], [17] that was rescued by heterologous expression of a *TCF/Pan* cDNA (Figure 6C). Mutation of a single cysteine residue (Mutant 4) abolished the ability of the transgene to complement the *TCF/Pan* mutant phenotype (Figure 6F). Given our findings that Mutant 4 is defective in binding to the Helper site in vitro (Figure 4), its lack of rescue indicate that the DNA-binding activity of the C-clamp is absolutely required for TCF/Pan’s function in patterning the embryonic epidermis of the fly.

### C-clamp Inhibition of HMG Domain DNA Binding

In our previous report characterizing TCF/Pan DNA binding, we noted that mutating the C-clamp domain resulted in increased binding to a HMG site probe [25]. Similar data has also been found in studies with different TCF4 isoforms, i.e., isoforms lacking the C-clamp had elevated binding to HMG site probes [43], [44], [45]. We have confirmed this “HMG inhibitory” effect using quantitative EMSA, finding that mutations in the C-clamp resulted in up to a five fold increase in binding to a HMG site probe (Figure 4B, 4D). Our finding that the HMG and C-clamp domains directly interact in vitro (Figure 5) provides a mechanism for the inhibitory effect: in the absence of a Helper site, the C-clamp binds to the HMG domain, interfering with its ability to bind to DNA.

**Figure 8:**
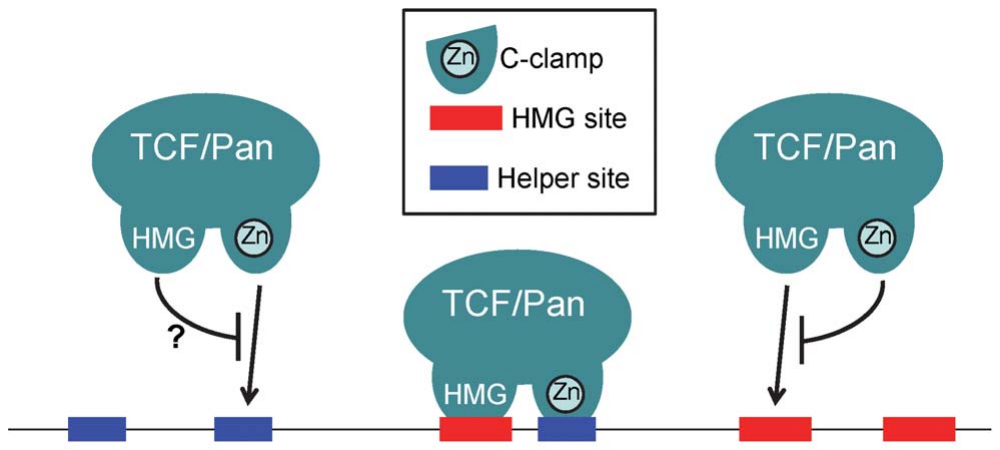
Model depicting a dual role for the C-clamp in enhancing the DNA-binding specificity of TCF/Pan. The presence of the HMG and C-clamp domains allows TCF/Pan to bind to HMG-Helper site pairs (middle). In addition, the C-clamp may inhibit TCF/Pan from binding to unpaired HMG sites (right). Conversely, the HMG domain may inhibit the C-clamp from binding unpaired Helper sites (left).

The inhibitory effect is potentially interesting because it could provide an additional mechanism for increasing TCF/Pan DNA-binding specificity. Due to the degeneracy in what constitutes a HMG binding site [21], the fly genome contains a large number of potential sites that TCF/Pan could bind to via its HMG domain, which could prevent it from locating bona fide W-CRMs [1]. The C-clamp likely helps to overcome this problem by promoting TCF/Pan binding to HMG-Helper site pairs, while the C-clamp-HMG domain interactions could also prevent binding of these domains to unpaired HMG and Helper sites (Figure 8).

Given that the C-clamp inhibits HMG domain-HMG site interactions, it is also possible that the HMG domain interferes with C-clamp binding to Helper site DNA (Figure 8). This interaction could explain why Helper site DNA is bound very poorly by recombinant HMG-C-clamp proteins [25], while binding of the Helper site by C-clamp alone is readily detectable (Figure 1). It should be noted that while the C-clamp is sufficient to bind to Helper sites, synthetic reporters containing up to 12 copies of a consensus Helper site are not activated by Wnt/ß-cat signaling [25]. This suggests that the C-clamp-Helper site interaction is not as strong as the HMG domain-HMG site binding, since high-density HMG site reporters are activated by the pathway [20], [25] (Figure 3D, 3E).

Our mutagenesis analysis indicated that the DNA-binding and inhibitory activities of the C-clamp are separable (Figure 4). This is further supported by our findings that pretreatment of the C-clamp with the metal chelator OPA abolishes its DNA binding activity (Figure 2E, 2F) while having no effect on binding to the HMG domain (Figure 5B). The ability to genetically separate the two activities allows us to test their functional relevance, which demonstrated that the C-clamp’s DNA binding activity was essential for Wnt gene regulation (Figure 6F). Mutant 8 (Figure 3A) was the best candidate for a specific inhibitory mutant, since it displayed normal DNA binding activity and was compromised for inhibitory activity (Figure 4B –4D). Interestingly, Mutant 8 had a reduced ability to rescue W-CRM reporter gene activation in cultured cells (Figure 3B), even when expressed at high levels (Figure 3C). However, this mutant was still functional in rescuing the *TCF/Pan* mutant cuticle phenotype in fly embryos (Figure 6G).

The finding that a Mutant 8 TCF/Pan is still functional in the fly embryo is disappointing, but there are some important caveats to consider. Although wild-type and mutant TCF/Pan proteins are expressed at similar levels (Figure 6H), this might still be higher than the endogenous concentration, allowing the mutant TCF/Pan to saturate non-functional HMG sites and still leave enough mutant TCF to bind functional W-CRMs. To address this, the rescue experiments were repeated with transgenic *TCF/Pan* lines which were expressed at significantly lower levels than the ones used in Figure 6, but full rescue was still observed (data not shown). Another consideration is that the defect in the inhibitory activity of Mutant 8 is only partial, e.g., compared to Mutant 5 (Figure 4D). Additional mutations were generated, but they all resulted in partial loss of Helper site binding activity, rendering them useless for testing the specific role of the inhibitory function. It is likely that additional structural data on the C-clamp-HMG domain complex will be needed to design suitable mutations to address the biological role of the HMG inhibitory function of the C-clamp.

In sum, our findings provide valuable new information about the biochemical properties of the C-clamp, and confirm its biological importance in Wnt/ß-cat signaling. Given that almost all invertebrate TCFs possess a conserved C-clamp, our data will be relevant to important family members such as POP-1 in *C. elegans* [52] and TCF in Hydra [53]. Likewise, the C-clamps in TCF1E and TCF4E isoforms probably have similar properties as we have uncovered in TCF/Pan, which likely contribute to the ability of these isoforms to activate specific Wnt targets [24], [26], [27], [45] and promote oncogenesis [24], [26]. Whether C-clamps from other proteins, e.g., GLUT4EF, can bind to the HMG domain of TCFs is another interesting question that requires further investigation.

## Materials and Methods

### Plasmids

The protein expression vector for *Drosophila* cell culture, pAc5.1/TCF/Pan-V5/His (pAc-TCF/Pan), has been described previously [25]. The Quikchange II mutagenesis kit (Stratagene) was used to generate the various C-clamp mutants in the pAc-TCF/Pan expression vector. pAc-Arm* and the luciferase reporters, pGL3-nkd-intE and pGL3-6xHMG, have been described previously [25], [41].

The protein expression vectors for EMSA were generated by cloning the region encoding the HMG domain and the C-clamp from the pAc-TCF/Pan constructs into the XmaI and SacI restriction sites of the pET52b(+) vector (pET) (Merck Millipore). pET-HMG and pET-C-clamp were generated by cloning the respective coding regions (residues 271 to 369 for the HMG domain; residues 363 to 408 for the C-clamp) into the same sites. The pET vector encodes a C terminus 10xHis tag, which was used for protein purification.

For transgenic flies, the *TCF/Pan* ORF was cloned from the pAc-TCF/Pan constructs into the KpnI and XbaI restriction sites of the pUAST vector. This vector contains a C terminal V5 tag, which was used to detect the protein for Western blots.

### *Drosophila* Cell Culture, RNAi Knockdown and Transient Transfection

Kc167 cells were cultured in *Drosophila* Schneider’s media with 10% FBS. For RNAi knockdown, cells were treated with 10 µg/1×10^6^ cells of double-stranded RNA (dsRNA) targeting the 3′UTR of wild-type TCF/Pan. After 4 days, cells were diluted to 1×10^6^ cells/mL and transfected with a plasmid mix containing pGL3-nkd-intE or pGL3-6xHMG, pAc-TCF/Pan (wild-type or mutant), S-188-cc-RLuc, a Renilla luciferase reporter [54] and pAc-Arm*. The TCF/Pan constructs contain a 3′UTR different from that in endogenous TCF/Pan, preventing them from being targeted by the dsRNA. After 3 days, cells were harvested for the Dual Luciferase reporter assay (Promega).

### EMSA

Gel shifts were carried out using the Lightshift Chemiluminescent EMSA kit (Pierce). IRDYE-700 tagged DNA probes and His-tagged recombinant TCF/Pan fragments, purified from *E.coli,* were used. Band signals in the gel were detected and quantified using the LI-COR Odyssey Infrared Imaging System. After background subtraction, the percentage bound was calculated as the signal in the shifted band/total signal in that lane. Signals for all mutants were then normalized to the wild-type protein.

### Metal Chelator Treatment and ICP-MS

Purified proteins in the EMSA binding buffer were incubated with 3.6 mM of 1, 10-orthophenanthroline (OPA) for 20 minutes at room temperature. The DNA probe was then added and the mixture was incubated for 20 minutes on ice, before being loaded into the gel. In the negative control, nuclease free water was used instead of OPA. For the rescue, OPA treated proteins were incubated with 100 µM of each of the salts for 10 minutes at room temperature before DNA was added.

Samples of His-TCF and His-HMG proteins were sent for testing metal content using inductively coupled plasma mass spectrometry (ICP-MS), which was carried out by Dr. Ted Huston (Dept. of Earth and Environmental Sciences, University of Michigan).

### Transgenic Flies

Transgenic fly lines were generated by BestGene Inc. using P-element mediated transformation. Crosses for rescue of the *TCF/Pan* embryonic phenotype were set up as indicated in Figure 6A. For the cuticle analysis, flies were allowed to lay eggs on grape juice plates for 6–8 hours at 25°C. Flies were then removed and plates were incubated for an additional 24–36 hours at 25°C. During this time a wet yeast paste was applied to the centre of each plate to attract hatching larvae and periodically removed leaving behind unhatched embryos. Unhatched embryos were collected and their cuticles were prepared as described previously [55].

For the Western blots, flies were allowed to lay eggs for 4 hours and the embryos were incubated for an additional 6 hours, all at 25°C. Embryos were collected and dechorionated. They were then treated with hot SDS buffer and manually ground for 5 minutes. Samples were loaded into a gel. TCF-V5 was detected using mouse anti-V5 antibody (1∶5000, Invitrogen). Tubulin was used as a loading control. The ECL kit (Pierce) was used to visualize the blots.

*TCF/Pan* mutant alleles used were *TCF^2^* and *TCF^3^. TCF^2^* contains a base pair loss of ATT to AT leading to a frameshift at I106 and *TCF^3^* contains a CAA to TAA mutation resulting in a stop codon at Q319 in the HMG domain [17].

### GST Pulldown Assay

2 µg of His-C-clamp and 2 µg of either GST or GST-HMG were incubated for 1 hour at 4°C with rotation in 20 mM Tris-HCl (pH 7.62), 150 mM NaCl and 1% Triton X-100 binding buffer. The mixture was then incubated with Glutathione Sepharose beads (GE Healthcare) for an additional 2 hours at 4°C with rotation. After 4–5 washes with binding buffer, the sample was treated with hot SDS buffer and loaded into a gel. His-C-clamp was detected using mouse anti-His antibody (1∶3000, GE Healthcare). Micrococcal nuclease treatment was carried out as described previously [56], using 6.6 units of micrococcal nuclease (Sigma) for a 200 µL reaction volume. In addition, the binding buffer used for this treatment contained 5 mM CaCl_2_. OPA treatment was carried out by incubating His-C-clamp in 0.363 µM OPA for 20 minutes at room temperature. GST or GST-HMG was then added and the mixture was incubated for 1 hour at 4°C with rotation prior to GST pulldown. In the negative controls for both treatments, nuclease free water was used instead of micrococcal nuclease or OPA.
