# Correction: Structure-Function Analysis of the C-clamp of TCF/Pangolin in Wnt/ß-catenin Signaling

**DOI:** 10.1371/journal.pone.0091378

**Published:** 2014-03-07

**Authors:** The PLOS ONE Staff

The name of the first author is incorrect. The correct name is: Aditi J. Ravindranath.

The correct Citation is: Ravindranath AJ, Cadigan KM (2014) Structure-Function Analysis of the C-clamp of TCF/Pangolin in Wnt/ß-catenin Signaling. PLoS ONE 9(1): e86180. doi:10.1371/journal.pone.0086180.
